# Public Awareness of Colorectal Cancer Screening in the Al-Baha Region, Saudi Arabia, 2022

**DOI:** 10.7759/cureus.32386

**Published:** 2022-12-10

**Authors:** Ali G Alghamdi, Zahraa Jumah A Almuhanna, Zainab Hussain M Bu Hulayqah, Fatimah Abdulaziz G Algharsan, Hashim A Alghamdi, Hadeel Mohammed A Alzahrani

**Affiliations:** 1 General and Colorectal Surgery, College of Medicine, Al-Baha University, Al-Baha, SAU

**Keywords:** cancer, saudi arabia, al baha, knowledge, awareness, screening, crc, colonoscopy, colorectal cancer

## Abstract

Background: According to the WHO, cancer is ranked as the second leading cause of death, accounting for an estimated 9.6 million deaths in 2018. This study aimed to assess public awareness about colorectal cancer (CRC) screening and the barriers that prevent Saudi individuals from undergoing CRC screening.

Materials and methods: A cross-sectional study was conducted in the Prince Mishari Bin Saud General Baljurashi Hospital, and a supervised self-administered questionnaire was utilized. Sociodemographic data, knowledge about colorectal cancer, and attitude toward screening were included in the survey. Data were analyzed using IBM SPSS Version 23 (SPSS Inc., Chicago, IL, USA) and Pearson's chi-square test. A P<0.05 was considered statistically significant.

Results: A total of 396 eligible participants completed the survey. About 209 (52%) were female, and 124 (31.3%) belonged to the age group of 18-29 years. Nearly (49.7%) knew that detecting colorectal cancer before symptoms appear is possible. About 64% of the participants cited colonoscopy as the screening method for CRC. More than half of the participants (58.1%) expressed their willingness to be screened for colorectal cancer, while only 2.8% reported that they had undergone screening before. Participants with higher educational status demonstrated better knowledge regarding CRC than others (p<0.05).

Conclusion: The overall knowledge of CRC was found to be poor in Al-Baha residents, irrespective of age. Implementing new strategies to increase public awareness about colorectal cancer will aid in the early diagnosis of CRC. We recommend targeted education and screening programs to improve the level of screening awareness and aid in the early diagnosis of colorectal cancer.

## Introduction

According to the WHO, cancer is ranked as the second leading cause of death, accounting for an estimated 10 million deaths in 2020. Moreover, about 70% of cancer deaths are evident in low and middle-income countries, mostly due to unattainable diagnosis and treatment or late-stage presentation as a consequence of deficient screening or awareness [1]. Colon cancer ranks first and third most common cancer among the Saudi male and female population, respectively, as reported by the Saudi Cancer Registry (SCR) 2015 Cancer Incidence Report [2]. It is also important to highlight that colorectal cancer (CRC) is the third leading cause of cancer-related deaths in the Saudi population [3]. The median age at which the diagnosis was made is 60 for Saudi men and 57 for Saudi women [4]. The principle of health promotion programs is to prevent diseases rather than treat them, and this goal is achieved by implementing screening programs. Screening is a secondary prevention that aims to detect diseases early and limit and halt their effects [5].

The American Cancer Society (ACS) recommends colorectal cancer screening to be started at the age of 45 years in average-risk asymptomatic men and women, with an earlier age and more frequent screening for high-risk individuals [6]. The Saudi national guidelines recommend screening to begin at the age of 45 for the Saudi population as a result of the variations in the median age of presentation. A colonoscopy every 10 years for average-risk individuals is the screening tool of choice in regard to colorectal cancer. Flexible sigmoidoscopy every five years when combined with an annual fecal immunochemical test (FIT), or every three years without an annual FIT, is another strongly recommended CRC screening tool [7]. Regular screening of CRC aids in the detection of precancerous lesions, such as polyps, but it is also crucial in finding cancerous lesions early and managing them accordingly. Early detection is the cornerstone of survival since CRC is highly treatable at early stages [8]. Other screening tests that might be used are computed tomographic colonography and a guaiac-based fecal occult blood test [9].

Risk factors for CRC can be classified as modifiable and non-modifiable. Modifiable risk factors include a sedentary lifestyle, obesity, low-fiber and high-fat diet, alcohol consumption, and tobacco use, while the non-modifiable risk factors include advanced age, inflammatory bowel disease, family history of CRC, and a genetic syndrome such as Lynch syndrome [10,11].

CRC can be asymptomatic, yet some complaints include a change in bowel habits, rectal bleeding, abdominal pain, fatigue, and unintentional weight loss [12]. The choice to undergo CRC screening primarily relies on an individual’s awareness of the disease’s risk factors and symptoms. Although some people might be aware of the screening recommendations, the embarrassment of being subjected to a colonoscopy may lead to their refusal to undergo one. Becoming acquainted with CRC screening guidelines and recommendations should, in turn, help in the early detection and treatment of CRC. For instance, a 2016 study conducted in Riyadh found that most respondents (42.9%) believe that screening should only begin at symptom onset [13]. Similarly, only 22.1% knew the correct time for CRC screening to begin in a 2018 study in Asir [14]. Likewise, a study done in Al-Madinah reported that only 13.1% of the participants correctly answered the appropriate age for screening, and only 20.8% are considering screening [15]. A recent study done in Makkah province showed that out of 97 participants with relatives diagnosed with CRC, only 21.6% had heard about CRC screening [16]. Even though many studies were conducted across the Kingdom of Saudi Arabia, no specific study has measured Al-Baha residents’ practices and awareness about CRC screening. Thus, this study aims to evaluate public awareness regarding CRC screening and implement new strategies to overcome the barriers that prevent Saudi individuals from undergoing CRC screening.

The primary study objectives were to assess the knowledge and awareness regarding CRC and its risk factors, and the attitudes toward CRC screening among Al-Baha residents, while also evaluating the relationship of this knowledge, awareness, and attitudes with sociodemographic characteristics.

## Materials and methods

A cross-sectional survey was conducted using a pretested questionnaire among individuals attending Prince Mishari General Hospital, Al-Baha region, Saudi Arabia. Residents of the Al-Baha region aged 18 years and above who were Arabic speakers were included in our study. Those who were non-residents of the Al-Baha region, aged less than 18 years, health care workers, and individuals with a history of CRC were excluded from this survey. The questionnaire was distributed among randomly picked Al-Baha region residents who were visiting Prince Mishari General Hospital (PMGH) at random hours during the weekdays. The data were collected in the duration of 60 days.

The primary version of the questionnaire was in English but was translated into an Arabic version for the need of data collection. The internal consistency was measured using Cronbach's alpha, and test-retest reliabilities between the initial and final questionnaires for each item were calculated using kappa statistics. A Cronbach's α value >0.7 was considered for our questionnaire to be internally consistent. An expert proficient in both English and Arabic then translated the English version into Arabic. Finally, the Arabic version was back-translated to the English version by another independent translator who was blind to the original English version. The principle investigator compared both the primary and back-translated versions of the questionnaire.

The questionnaire included three sections; the first part included sociodemographic data (age, gender, education level, occupation, residence, and marital status) (Appendix 1, Table 6); the second part had items that assessed the knowledge and awareness of participants about colorectal cancer (Appendix 2, Table 7); and the third part included items used to assess the attitude of participants toward available CRC screening methods (Appendix 3, Table 8).

A minimum sample size was calculated using the Richard Geiger equation at a confidence level of 95% and 80% power [17]. A minimum sample of 328 was found appropriate for our study, and the final sample size acquired was 511. The data collection was conducted for a duration of two months.

Statistical analysis and data management

All the collected responses were tabulated on a Microsoft Excel sheet (Microsoft Corporation, Redmond, WA) and the responses were coded and transferred to the IBM Statistical Package for Social Sciences, Version 23 (SPSS Inc., Chicago, IL, USA) for data analysis. The data analysis was performed by an independent biostatistician. Descriptive statistics in the form of frequencies and percentages using suitable tables and figures were used to represent categorical data. Pearson's chi-square test for association between categorical variables was used, and a p-value of <0.05 was considered to be statistically significant.

## Results

**Figure 1:**
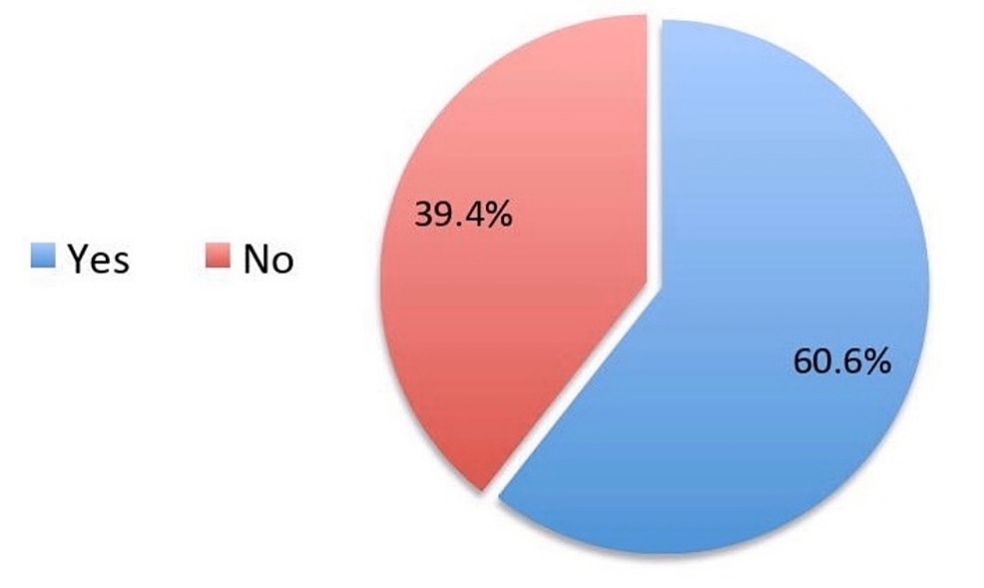
Have you heard about colorectal cancer?

**Figure 2:**
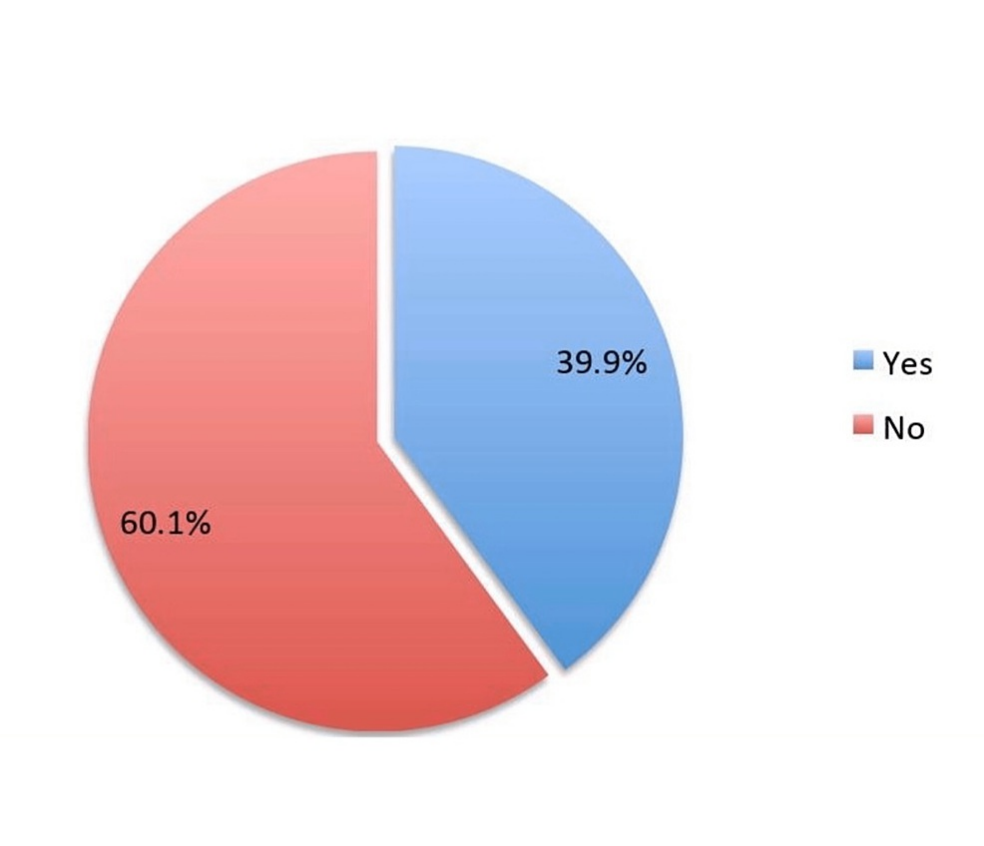
Have you heard about colorectal screening?

The study survey received around 511 responses, of which we included only 396 participants who satisfied our study eligibility criteria. The sociodemographic analysis showed that 209 (52.8%) were females, 124 (31.3%) belonged to the age group of 18-29 years, 226 (57.1%) had a bachelor's level of education, 165 (41.7%) were employed, and 257 (64.9%) were married (Table 1). Assessment of colorectal cancer showed that 240 (60.6%) participants have heard about it (Figure 1). The responses related to knowledge about colon and colorectal cancer and the correct answers are given in Table 2. About 64.6% agreed that the colon is a large intestine. Only 23% and 29.3% knew that water absorption and waste storage are the functions of the colon, respectively. About 11.4% knew that colorectal cancer has a "high" prevalence, and only 23.7% knew that males are more likely to develop this type of cancer. The most commonly cited risk factors were smoking (55.8%), followed by colonic polyps (44.2%), advanced age (42.4%), eating foods rich in red meat and poor in fiber (41.9%), obesity (39.6%), and inflammatory bowel diseases (36.6%). When we asked about the symptoms of colorectal cancer, about 60.9% mentioned abdominal pain, followed by abdominal swelling (46.7%), blood in the stool or dark-colored stools (46%), persistent changes in bowel movements (41.2%), unexplained weight loss (40.9%), and nausea and vomiting (34.6%). Nearly 49.7% knew that it is possible to detect colorectal cancer before symptoms appear, whereas only 29% knew that detection of colorectal cancer should begin at the age of fifties. However, 67.7% knew that colorectal cancer could be treated when detected at an early stage. It was found that only 39.9% of the participants were aware of the colorectal cancer screening (Figure 2).

**Table 1:** Sociodemographic details (n=396).

		N	%
Gender	Female	209	52.8
	Male	187	47.2
Age (years)	18-29	124	31.3
	30-39	111	28.0
	40-49	96	24.2
	50-59	40	10.1
	≥60	25	6.3
Education level	Primary	13	3.3
	Middle	19	4.8
	Secondary	67	16.9
	Diploma	27	6.8
	Bachelors	226	57.1
	Postgraduate	17	4.3
	Uneducated	27	6.8
Occupation	Employee (any job other than a health practitioner)	165	41.7
	Retired	34	8.6
	Student	70	17.7
	Unemployed	127	32.1
Marital status	Unmarried	113	28.5
	Married	257	64.9
	Divorced	22	5.6
	Widower	4	1.0

**Table 2:** Responses related to knowledge items related to colon and colorectal cancer. *Correct answers.

		N	%
The colon is part of	Large intestine*	256	64.6
	Small intestine	39	9.8
	Stomach	39	9.8
	I do not know	62	15.7
Function of colon	Waste storage*	116	29.3
	Water absorption*	91	23.0
	Digestion of food	90	22.7
	No particular function	5	1.3
	I do not know	94	23.7
How common is colon cancer?	High*	45	11.4
	Average	131	33.1
	Rare	77	19.4
	I do not know	143	36.1
What is the group most at risk of developing colorectal cancer?	Female	50	12.6
	Male*	94	23.7
	Both genders	152	38.4
	I do not know	100	25.3
What are the risk factors that increase the likelihood of developing colorectal cancer?	Advanced age*	168	42.4
	Sedentary lifestyle*	156	39.4
	Obesity*	157	39.6
	Eating foods rich in red meat and poor in fiber*	166	41.9
	Smoking*	221	55.8
	Inflammatory bowel diseases*	145	36.6
	Colon polyps*	175	44.2
	Genetical*	134	33.8
	I do not know	60	15.2
What are the symptoms of colorectal cancer? (choose all that apply)	Abdominal swelling*	185	46.7
	Abdominal pain*	241	60.9
	Nausea and vomiting*	137	34.6
	Unexplained weight loss*	162	40.9
	Persistent change in bowel movements (diarrhea or constipation)*	163	41.2
	Blood in the stool or dark-colored stools*	182	46.0
	Yellowing of the eyes and skin*	47	11.9
	No symptoms	54	13.6
	I do not know	73	18.4
Do you think it is possible to detect colorectal cancer before symptoms appear?	Yes*	197	49.7
	I do not know	78	19.7
	No	121	30.6
At what age does the detection of colorectal cancer begin?	20s	44	11.1
	50s*	115	29.0
	70s	37	9.3
	Only when there are symptoms	95	24.0
	I do not know	105	26.5
In your opinion, can colorectal cancer be treated when detected at an early stage?	Yes*	268	67.7
	I do not know	49	12.4
	No	79	19.9

**Figure 3:**
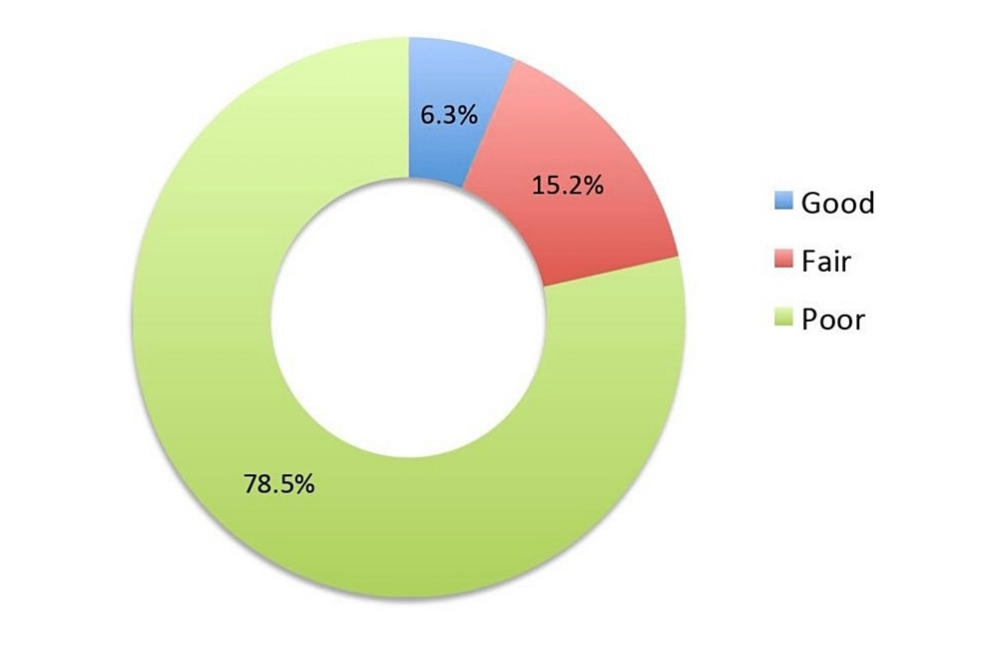
Overall knowledge regarding colon and colorectal cancer.

The total knowledge level related to colon and colorectal cancer was calculated by totaling the correct response(s) for each knowledge item. A score of "1" was given to the correct answers for items with one correct answer, whereas items that had multiple correct answers were given 0.50 for each correct response. The total knowledge scores were calculated and then categorized based on the percentage of scores obtained, whereas a score of ≥75% was considered as "good," 60-74.9% as "fair," and <60% as "poor." It was found that only 6.3% of the participants demonstrated a "good" knowledge level, whereas the majority (78.5%) had "poor" knowledge levels (Figure 3).

Assessment of the relationship between participants' level of knowledge and sociodemographic characteristics showed that there were no statistically significant differences observed for gender (p=0.093), age (0.711), and marital status (p=0.785). However, educational level and occupation showed significant associations with participants' knowledge levels. It was observed that participants who had postgraduate and bachelor's education demonstrated significantly more "good" knowledge levels than others (p<0.001). Also, those who were employed significantly demonstrated more "good" knowledge levels than others (p=0.006) (Table 3).

**Table 3:** Relationship between knowledge level and sociodemographic characteristics. *P-value for Pearson chi-square test (a p-value <0.05 is considered statistically significant).

			Knowledge level			Total	P-value*
			Good	Fair	Poor		
Gender	Female	N	11	25	173	209	0.093
		%	5.3%	12.0%	82.8%	100.0%	
	Male	N	14	35	138	187	
		%	7.5%	18.7%	73.8%	100.0%	
Age	18-29	N	9	16	99	124	0.711
		%	7.3%	12.9%	79.8%	100.0%	
	30-39	N	8	13	90	111	
		%	7.2%	11.7%	81.1%	100.0%	
	40-49	N	5	20	71	96	
		%	5.2%	20.8%	74.0%	100.0%	
	50-59	N	1	7	32	40	
		%	2.5%	17.5%	80.0%	100.0%	
	≥60	N	2	4	19	25	
		%	8.0%	16.0%	76.0%	100.0%	
Education level	Primary	N	0	0	13	13	<0.001
		%	0.0%	0.0%	100.0%	100.0%	
	Middle	N	0	1	18	19	
		%	0.0%	5.3%	94.7%	100.0%	
	Secondary	N	3	6	58	67	
		%	4.5%	9.0%	86.6%	100.0%	
	Diploma	N	1	4	22	27	
		%	3.7%	14.8%	81.5%	100.0%	
	Bachelors	N	16	44	166	226	
		%	7.1%	19.5%	73.5%	100.0%	
	Postgraduate	N	5	4	8	17	
		%	29.4%	23.5%	47.1%	100.0%	
	Uneducated	N	0	1	26	27	
		%	0.0%	3.7%	96.3%	100.0%	
Occupation	Employee (any job other than a health practitioner)	N	13	36	116	165	0.006
		%	7.9%	21.8%	70.3%	100.0%	
	Retired	N	3	7	24	34	
		%	8.8%	20.6%	70.6%	100.0%	
	Student	N	4	9	57	70	
		%	5.7%	12.9%	81.4%	100.0%	
	Unemployed	N	5	8	114	127	
		%	3.9%	6.3%	89.8%	100.0%	
Marital status	Unmarried	N	17	42	198	257	0.785
		%	6.6%	16.3%	77.0%	100.0%	
	Married	N	8	14	91	113	
		%	7.1%	12.4%	80.5%	100.0%	
	Divorced	N	0	3	19	22	
		%	0.0%	13.6%	86.4%	100.0%	
	Widower	N	0	1	3	4	
		%	0.0%	25.0%	75.0%	100.0%	

Asking the participants about methods used for early detection of colorectal cancer that they have heard about revealed that the most commonly cited method was colonoscopy (64%), followed by CT scan (37.1%) and barium study (36.9%). About 14.4% reported that they have relatives who have been diagnosed with colorectal cancer. More than half (58.1%) agreed that they are ready to get early screening for colorectal cancer, even without symptoms. Only 2.8% reported that they had done early detection of colorectal cancer. The most common source of information on colon and colorectal cancer among participants was the internet (42.7%), followed by social networking sites (37.1%), relatives or friends (24%), and school or university (37.1%) (Table 4).

**Table 4:** Attitudes and perceptions related to colorectal cancer.

		N	%
Early detection of colorectal cancer methods already knew	Colonoscopy	257	64
	Fecal occult blood test	134	33.8
	Ultrasound sonography	78	19.7
	Barium study	146	36.9
	CT scan	147	37.1
	X-ray	93	23.5
	I haven't heard of any of these methods	74	18.7
Have relatives diagnosed with colorectal cancer	Yes	57	14.4
	I do not know	42	10.6
	No	297	75.0
Ready to get early screening for colorectal cancer even without symptoms	Yes	230	58.1
	No	166	41.9
Done early detection of colorectal cancer	Yes	11	2.8
	No	385	97.2
Source information on colon and colorectal cancer	Health practitioner (doctor, nurse, etc.)	55	13.9
	School/university	84	21.2
	Social networking sites	147	37.1
	TV/radio	71	17.9
	Awareness campaign	42	10.6
	Relatives or friends	95	24.0
	Internet	169	42.7

It was observed that participants who had relatives diagnosed with colorectal cancer comparatively demonstrated more "good" knowledge levels than others who didn't have any (p=0.011). Also, it was found that participants who demonstrated a "good" knowledge level were the ones who were ready to get early screening for colorectal cancer even without symptoms (p<0.001). Participants who have done early detection of colorectal cancer significantly demonstrated more "good" knowledge levels than others (p<0.001) (Table 5).

**Table 5:** Relationship between knowledge level and attitudes toward colorectal cancer. *p-value for Pearson chi-square test (a p-value <0.05 is considered statistically significant).

			Knowledge level			Total	P-value
			Good	Fair	Poor		
Have relatives diagnosed with colorectal cancer	Yes	N	7	15	35	57	0.011
		%	12.3%	26.3%	61.4%	100.0%	
	I do not know	N	1	4	37	42	
		%	2.4%	9.5%	88.1%	100.0%	
	No	N	17	41	239	297	
		%	5.7%	13.8%	80.5%	100.0%	
Ready to get early screening for colorectal cancer even without symptoms	Yes	N	23	49	158	230	<0.001
		%	10.0%	21.3%	68.7%	100.0%	
	No	N	2	11	153	166	
		%	1.2%	6.6%	92.2%	100.0%	
Done early detection of colorectal cancer	Yes	N	4	3	4	11	<0.001
		%	36.4%	27.3%	36.4%	100.0%	
	No	N	21	57	307	385	
		%	5.5%	14.8%	79.7%	100.0%	

## Discussion

This study's findings suggest a lack of public awareness about CRC screening in Saudi Arabia. This is in agreement with the findings of a previous study that showed a similar issue [16,17]. Males were more knowledgeable about CRC and screening when compared to females. However, there was no statistically significant gender difference observed in their knowledge about the function of the colon. Furthermore, there is no significant relationship between gender and screening for CRC. It is in contrast to the Knudsen et al. study that reported that women are more likely to be interested in and actively seek out health and disease prevention information, which is consistent with previous studies [18]. It should be noted that our research population does not reflect the demographics of Saudi Arabia as a whole since all of our participants were recruited from a single tertiary medical center in Al-Baha, and over half of them had completed a bachelor’s degree or a higher educational level. Thus, we could have expected lower rates of awareness if our study had included people from more rural locations.

It was found that participants who had relatives diagnosed with CRC demonstrated comparatively better knowledge levels than others. This is consistent with previous studies that showed that knowledge of CRC risk factors and warning signs was higher among people who had personal experience with the disease [19-21]. Our findings showed that only 2.8% of the participants had previously undergone colorectal cancer screening, and 58.1% of the participants reported that they were ready to screen for CRC. The screening rate reported in our study was lower compared to a previous study done in Saudi Arabia, which reported a screening rate of 8.6% [22]. 

One of the most significant obstacles to CRC screening is a lack of awareness about the need for screening [21,23]. There is evidence that primary care physicians have a significant role in promoting CRC screening and raising knowledge of screening recommendations [20]. It is reported that a lack of strong physician recommendations was a factor in the low screening rates because it prevented individuals from talking to their doctors about health concerns [24]. Individuals' adherence to screening is also likely to rise if they hear from friends, relatives, or co-workers who have been cured of colorectal cancer [25]. No statistically significant relationship between the gender of participants and history of CRC and being ready for CRC screening was observed. This is similar to a previous study done by Carnahan et al., which reported that only a few people knew about the CRC screening guidelines with no gender differences [26]. Another study done in Qatar by Al-Dahshan et al. reported a poor correlation between age and educational level when assessing CRC awareness level [27]. There was also no statistically significant difference observed between the age of the participants and their readiness to get CRC screening. Recognizing who should get CRC screening before the age of 45 due to a strong family history of CRC or an inherited cancer syndrome is crucial [28]. Unfortunately, we did not assess our participants' risk of getting CRC in our study. The significance of family history-based risk stratification is demonstrated by the fact that almost one-quarter of individuals with early onset CRC could have been candidates for early screening [29]. Higher educational levels were associated with better knowledge about the various aspects of screening. Additionally, the study showed that married respondents exhibited superior knowledge about CRC and its screening in comparison to other participants with a different marital status, which is consistent with the findings of a previous study [4]. Furthermore, the majority of the participants knew about the methods of early detection of CRC, and most of them chose colonoscopy as the screening method. Similar findings were found in other studies conducted in other provinces of Saudi Arabia [4,13]. We strongly encourage the strategic adoption of techniques to ensure equity in access across populations and expand screening practices in Saudi Arabia. This provides an opportunity to mitigate the effects of CRC after the age of 45, but it should also serve as a reminder to spread the benefits as widely as possible. According to our findings, the majority of participants learned about CRC via online resources, including the Internet and social media. The implementation of planned awareness campaigns and the adoption of an awareness month can improve the public's familiarity with CRC. Promoting these events on television and digitally can help attract people of a wider variety of ages and socioeconomic backgrounds.

This study has several limitations, and this should be borne in mind before interpreting the findings. Firstly, the sample size was small, and one-third of the sample belonged to the age group of 18-29, as this age group had a considerably lower prevalence of CRC. Secondly, the participants' economic status and distribution between rural and urban areas were unknown. Thirdly, as we adopted a self-administered questionnaire, this could have led to recall bias and social desirability bias. Therefore, future research should concentrate on these elements in order to more precisely measure people in need of educational initiatives.

## Conclusions

CRC is the most common cancer among Saudi males, and its mortality rate is high if diagnosed late. The overall knowledge of CRC was found to be poor in Al-Baha residents, irrespective of age. More than half of the study population had not heard about CRC screening, and almost half of the participants were not willing to undergo CRC screening, which might be attributed to a low level of awareness. Implementing new strategies to increase public awareness about colorectal cancer will aid in the early diagnosis of CRC. We recommend targeted education and screening programs to improve CRC screening awareness.

## Appendices

Appendix 1

**Table 6:** Sociodemographic data.

Residency area	Al-Baha Region, Southern region
	Southern region
	Central region
	Eastern region
	Northern region
	Western region
Gender	Female
	Male
Age	Below 18 years
	18-29 Years
	30-39 Years
	40-49 Years
	50-59 Years
	60 Years and above
Education level	Elementary school
	Middle school
	High school
	Academic
	Other
Occupation	Student
	Unemployed
	Healthcare worker
	Engineer
	Business
	Other (please specify)
Marital status	Single
	Married/engaged
	Divorced
	Widowed

Appendix 2

**Table 7:** Knowledge and awareness about colorectal cancer. CRC: colorectal cancer.

Have you ever heard of colorectal cancer?	Yes
	No
	I don’t know
The colon is part of	Large intestine
	Small intestine
	The stomach
	I don’t know
Colon function is	Digestion of food
	Waste storage
	Water reabsorption
	Does not have a function
The incidence of colorectal cancer is	High
	Average
	Rare
	I don’t know
Who is more likely to develop colorectal cancer	Males
	Females
	Both equally
	I don’t know
Check from the following what you think might be a risk factor for CRC: (check all that applies)	Old age
	Sedentary lifestyle
	Obesity
	Low fiber diet and high in red meats
	Smoking
	Inflammatory bowel disease
	Polyps in the colon
	Hereditary factors
	I don’t know
Check from the following what you think might be a symptom of CRC: (check all that applies)	Abdominal tumor
	Abdominal pain
	Nausea and vomiting
	Unintentional weight loss
	Fatigue without any cause
	Changes in bowel habits (constipation or diarrhea)
	Blood in the stool or dark stool
	Yellow discoloration of eyes and skin
	Can develop without obvious symptoms
	I don’t know

Appendix 3

**Table 8:** Awareness and attitude toward CRC available screening methods. CRC: colorectal cancer.

Have you ever heard about CRC screening before?	Yes
	No
	I don’t know
Do you think CRC can be detected early before the onset of symptoms?	Yes
	No
	I don’t know
When does screening for CRC start:	20s of age
	50s of age
	70s of age
	Only when symptoms develop
	I don’t know
Do you think CRC can be treated if detected early?	Yes
	No
	I don’t know
Which of the following screening methods do you know to be used in the early detection of CRC: (check all that applies)	Fecal occult blood test
	Colonoscopy
	X ray
	CT scan
	Barium study
	Ultrasound
	I don’t know
Do you have any relative diagnosed with colorectal cancer?	Yes
	No
	I don’t know
Were you diagnosed with colorectal cancer?	Yes
	No
Are you willing to screen for colorectal cancer even without symptoms?	Yes
	No
Have you gone through colorectal cancer screening before?	Yes
	No
If yes, how many times, and what was the tool?	By your healthcare worker
	From your studies
	The web
	Social media
	TV and/or radio
	Awareness campaign
	Relatives and friends
	No source
